# Public health literacy unsold during panic buying

**DOI:** 10.1016/j.amsu.2022.104156

**Published:** 2022-07-12

**Authors:** Sheikh Shoib, Aisha Lodi, Amna Saleem, Aishatu Yusha'u Armiya'u, Serkan Turan, Sharad Philip

**Affiliations:** Department of Psychiatry, Jawahar Lal Nehru Memorial Hospital, Srinagar, Kashmir, India; Jinnah Medical and Dental College, Karachi City, Sindh, 74800, Pakistan; Department of Psychiatry, College of Medical Sciences Abubakar Tafawa Balewa University, Bauchi state, Nigeria; Department of Child and Adolescent Psychiatry, Bursa Uludag University Medical Faculty, Turkey; Department of Psychiatry, NIMHANS, Bangalore, India

## 1 Introduction

COVID 19 lockdowns and mobility restrictions and an infodemic have led to panic buying globally [1]. Panicked consumers bought cleaning and hygiene products, food, house-keeping materials and medicines [2]. Because the COVID-19 epidemic is still in its early stages, panic buying is a common occurrence that has just reawakened public attention. This leads to scarcity of goods and price inflation-maximally impacting lower socio-economic strata. Its relationship with public health literacy remains unexamined. This commentary aims to elaborate the role of health literacy in mitigating panic buying. An unprecedented crisis, lack of reliable information and general fear and uncertainty drove panic buying. The differences between the haves and the have-nots, the physically able and the physically disabled, the young and the old are highlighted during times of crisis. But, as we see in many Western countries, panic buying is not limited to grocery stores and pharmacies. It also poses a severe danger to continuing worldwide efforts to achieve Sustainable Development Goal 3.8 by 2030, which calls for “universal access to safe, effective, high-quality, and affordable critical medicines and vaccines” [3]. The research on the effects of panic buying is slightly more robust. Arafat et al. (2020) analysed 784 media reports from 93 countries on responsible factors for panic buying and reported that 78% was due to sense of scarcity, 66.07% increase demands, 45.02% importance of the product, 23.33% hike anticipation and 13.215 due to COVID-19 factors [4]. Panic buying feeds into further panic buying by causing a scarcity of basic supplies. From a public health perspective, behavioural and psychological explanations and interventions become extremely relevant [5]. The goal of this letter is to fill a research gap by combining the theoretical contributions of the health belief model, perceived scarcity, and expected regret theories to better understand the factors that influence panic buying behavior and their interrelationships.

### 1.1 Behavioural theories

Panic buying also known as “Stockpiling” [6] is a paradigm of discrete behavioural changes that prevails when community members purchase abnormal quantities of goods around the time a disaster is anticipated. Associated phenomena include a feared shortage of supplies and unprecedented surges in costs [7]. COVID- 19 has seen starkly dissimilar levels and trends in panic buying relative to other crises like floods and hurricanes [6]. A major dissimilarity includes differences in the extents of planning before shopping.

Compensatory Control theory (CCT) posits an experience of diminished control subsequent to pandemic related perturbation, unreliability and terror [4]. Cognitive distortions may provide a confusing overlap with hoarding disorder. Panic buying must be differentiated from compulsive buying disorders which is largely driven by the obsessive-compulsive shopping behaviours [7].

### 1.2 Understanding from psychology

Psychological causes of panic buying are grouped according to four main aspects [7]: community-based psychological determinants that contemplates the buying behaviour of a person's social alliance [6], insight of a person on menace of crisis and shortage of the basic commodities [8], dismay of the unknown due to emotions and inconstancy [4], imitating manners provoked by scarceness of self-control [8].

Additionally, poor public health messaging and information system and canards propagating a general mistrust of administrators may also lead to panic buying [4]. At times, frenzied buying is goaded on by end of season/clearance sales-at such times, contexts are different with no proceeding health or wellbeing related triggers [7]. At times, there may be altruistic motives especially when countries have poor governance [7].

## 2 How health literacy affects panic buying

Health literacy can be understood as being able to obtain and translate information towards maintaining and improving health [9]. Low levels of health literacy imply a lack of awareness or a general inability to translate health related information into appropriate action. Thus, in times of crises, health illiterate masses are more likely to misunderstand the situation and act inappropriately such as panic buying. Conflicting and contradicting messages from community leaders and media further compound the problem by portraying inconsistency, stoking alarm and undermine health messaging. They are also prone to misinformation and/or disinformation [10]. General literacy rates associate with health literacy-lower-middle-income countries (LMIC) with low levels of literacy include Iraq, Nigeria, Afghanistan, Guinea, Central African Republic and Chad [11]. Population in these countries may be most vulnerable to panic buying.

Panic buying impacts the entire population-causing inequitable distribution of resources [12]. Health literacy will protect persons from succumbing to anxieties and canards regarding shortfalls. This allows for every community member to have access needed resources, even during crises. Persons may misjudge severity and/or duration of crises. Aside from items of daily use people may stockpile medical supplies without realizing public health system's functioning or the impact of their stockpiling [13].

### 2.1 Recommendation

Crises tend to gum up supply chains and precipitate shortages - this makes resources costlier - increasing the inequity in resources availability. Health literacy measures ought to be introduced during schooling and education. Parents can be involved in such information sharing meetings so as to have maximum dissemination of health-related information. Such information sharing must continue in consistent and confidence inspiring manner even during a crisis. Public health administrators must also emphasize on such community health measures periodically. Community members must have adequately opportunities to clarify directly from health professionals-alongside health administrators must proactively seek out and dispel any misinformation. Media must act responsibly and serve to disseminate information consistently. Policymakers and practitioners need to aim for a better understanding of panic consumption patterns. Leaner business models and innovative practices for integrating various stakeholders should be designed to ensure continued supply even during crises. A noteworthy example of community led health messaging in a low resource setting is of women canvassers in Afghanistan who created about 4000 self-help groups and video messages on general health and hygiene [14]. Such health messaging models can help to improve health literacy. During the COVID-19 pandemic some countries addressed the detrimental impact of panic buying by rationing essentials items and modification of opening times so as to prioritize for the vulnerable population which includes elderly and healthcare workers which was commendable [3]. Lastly policy intervention may be necessary for the protection of citizens with limited mobility and whose households regularly run out of food items before the end of the month or those who can only afford to stock their homes when prices are lower.

## Ethical approval

No ethics approval was sought, because no humans or animals participated in this subject. Ethics approval or informed consent requirement were therefore not deemed to be applicable.

## Sources of funding

No funding

## Author contributions

**Sheikh Shoib**: Conceptualization, Methodology, Investigation, Writing - Original draft preparation, Project administration;

**Aisha Lodi**: Methodology, Investigation, Writing - Original draft preparation, Project administration;

**Amna Saleem**: Methodology, Investigation, Writing - Original draft preparation, Project administration;

**Aishatu Yusha'u Armiya'u**: Conceptualization, Methodology, Investigation, Writing - Original draft preparation.

**Serkan Turan**: Conceptualization, Methodology, Investigation, Writing - Original draft preparation, Project administration;

**Sharad Philip**: Methodology, Writing - Original draft preparation, Project administration.

## Registration of research studies

Name of the registry:
Unique Identifying number or registration ID:
Hyperlink to your specific registration (must be publicly accessible and will be checked):

## Guarantor

Sheikh Shoib.

## Consent

No written consent was sought, because no humans or animals participated in this subject. Ethics approval or informed consent requirement were therefore not deemed to be applicable.

## Data availability statement

Not applicable.

## Provenance and peer review

Not commissioned, externally peer reviewed.

## Declaration of competing interest

No conflict of interest declared.
